# Folate Status and Mortality in US Adults With Diabetes: A Nationally Representative Cohort Study

**DOI:** 10.3389/fcvm.2022.802247

**Published:** 2022-04-25

**Authors:** Hui Xiong, Xiaoxiao Li, Shuxian Cheng, Pengyu Chen, Sixu Guo, Xianli Huang, Yu Lu

**Affiliations:** ^1^Cardiac Function Department, Wuhan Wuchang Hospital, Wuchang Hospital Affiliated to Wuhan University of Science and Technology, Wuhan, China; ^2^Department of Cardiovascular Medicine, School of Clinical Medicine, Wuhan University of Science and Technology, Wuhan, China; ^3^Department of Cardiology, Wuhan Wuchang Hospital, Wuchang Hospital Affiliated to Wuhan University of Science and Technology, Wuhan, China

**Keywords:** RBC folate, diabetes, cardiovascular disease, cohort study, national survey

## Abstract

**Background:**

Public health concerns have gradually shifted from inadequate intakes to potential adverse effects associated with excessive folate intakes following the full implementation of mandatory folate fortification. This study aimed to examine the associations of red blood cell (RBC) folate with all-cause and cardiovascular disease (CVD) mortality among patients with diabetes.

**Methods:**

Data of 15,514 adults aged 20 years or older, who participated in the National Health and Nutrition Examination Survey (1988–1994), were analyzed as the baseline examination. The participants were linked to mortality data from the survey date until December 31, 2015. The associations of RBC folate with all-cause and CVD mortality were examined using multivariable Cox regression models.

**Results:**

During 297,708 person–years of follow-up (median of 19.2 years), 6,106 total deaths occurred, including 1,867 deaths from CVD, 1452 deaths from ischemic heart disease, and 415 deaths from stroke disease. The participants with the highest quartile of RBC folate had higher odds of diabetes (fully-adjusted odds ratio: 1.94 [95% CI: 1.53–2.48]). In Cox regression analyses, compared with the participants with the lowest quartile of RBC folate for diabetes, those from quartile 3 and quartile 4 had HRs (95% CIs) of 1.12 (0.87, 1.43) and 1.30 (1.04, 1.63) in all-cause mortality, respectively; in CVD mortality, the HRs were 1.73 (1.08, 2.76) and 1.47 (0.98, 2.22); in ischemic heart disease mortality, they were 2.01 (1.19, 3.39) and 1.62 (1.05, 2.50), respectively. However, high levels of RBC folate were negatively associated with all-cause mortality, CVD mortality and ischemic heart disease mortality in non-diabetes.

**Conclusion:**

From the nationally representative data, increasing levels in RBC folate were independently associated with an increased risk of all-cause and CVD mortality among those diagnosed with diabetes, but high levels of RBC folate had a mild protective effect in non-diabetes. The underlying mechanism regarding folate and adverse outcomes in diabetes warrants further clarification.

## Introduction

As one of the natural B vitamins, folate is widely found in staples, such as dark green vegetables, legumes, nuts, and fresh fruit (1, 2), or in the synthetic form used in supplements and food fortification programs (3). Folate is known to play an essential role in preventing neural tube defects (4). In 1998, the U.S. fully implemented the addition of folic acid to cereal grain (5). Subsequently, public health concerns have gradually shifted from inadequate intakes to potential adverse effects associated with excessive folate intakes (6).

Cardiovascular disease (CVD) is one of the most prominent global public health challenges in developed and developing countries in the 21st century (7). A meta-analysis suggested that folic acid contributes to lowering the risk of stroke and overall CVD events (8). However, some clinical research indicated that high-dose folic acid treatment provides no additional benefit on improving vascular function (9), and it does not alter markers of endothelial cell damage (10). A cohort study by Twum et al. (11) demonstrated that high folate levels are significantly associated with an increased risk of CVD among adults with hypertension. Recently, a cross-sectional study also concluded that elevated red blood cell (RBC) folate concentration is associated with increased odds of coronary heart disease in diabetes (12). The effect of high folate levels on CVD prevention among different metabolic populations has been disputed.

As one of the most common metabolic disorders in the world, diabetes has a great influence on macrovascular and microvascular systems, including CVD, diabetic kidney disease, retinopathy, and neuropathy (13). Current literature on the effect of folic levels on cardiovascular disease among patients with diabetes is limited. Thus, the data of diabetes from the National Health and Nutrition Examination Survey (NHANES) III, a nationally representative cohort, was analyzed in this study to examine the association of RBC folate with all-cause and CVD mortality.

## Methods

### Study Population

This study used data from NHANES III (1988–1994), which was conducted by the National Center for Health Statistics (NCHS), who used a stratified, multistage design to obtain a nationally representative sample of the non-institutionalized US population.

Diagnosed diabetes was defined as self-report of diabetes diagnosis by a physician or other health professional. Undiagnosed diabetes was defined as having a fasting plasma glucose level of 126 mg/dL or more or HbA1c level of 6.5% or more among individuals without diagnosed diabetes. Diabetes included both diagnosed and undiagnosed diabetes (14). A total of 33,994 participants were recruited to NHANES III between 1988 and 1994. Those who were younger than 20 years (*n* = 722) and pregnant (*n* = 288) at baseline were excluded in this study. Those with missing data on RBC folate (*n* = 760) were further excluded from analyses (Supplementary Figure 1). Finally, 2,972 patients with diabetes and 12,542 diabetes-free adults were included. The NCHS's Institutional Review Board reviewed and approved NHANES, and all participants provided a written informed consent.

### Assessment of RBC Folate

RBC folate reflects the folate turnover over the preceding 2–3 months and indicates tissue folate status; therefore, RBC folate is used rather than serum folate to assess long-term folate status in humans (15). Whole blood samples, which were frozen at or below −20°C and transported on dry ice, were measured to test RBC folate concentrations. The Centers for Disease Control and Prevention (CDC) used Quanta-phase I folate radioassay kit (Bio-Rad Laboratories) in NHANES III (1988–1991) and Quanta-phase II folate radioassay kit in NHANES III (1991–1994) to analyze RBC folate. Consistency of the two methods were confirmed by a series of quality control tests performed by CDC (16). The total participants were divided into four groups: RBC folate levels < 121 ng/mL (384.8 nmol/L) as lowest quartile; 122–161 ng/mL (388.0–512.0 nmol/L), 162–225 ng/mL (515.2–715.5nmol/L), and ≥ 226 ng/mL (718.7 nmol/L) as highest quartile.

### Assessment of Outcomes

A total of 12 identified information, including social security number, sex, and date of birth, were used to link the NHANES III participants with the National Death Index to ascertain vital status and cause of death. Participants were lost to follow-up with insufficient information on these matching criteria. The NCHS introduced a specific matching methodology in 2013, and further details are available on this website (17). The number of person–years of follow-up from NHANES III (1988–1994) was followed up from the date of examination to December 31, 2015, or the date of death, whichever occurred first. The average follow-up time in this study is 19 years, with a maximum of 27 years. UCOD_113 was created to conduct mortality analyses that span across years by using ICD-9 and ICD-10 coding. Death was classified as having CVD with an UCOD_113 code of 001 or 005 (18). CVD mortality included acute rheumatic fever and chronic rheumatic heart diseases (I00–I09), hypertensive heart disease (I11), hypertensive heart and renal disease (I13), ischemic heart diseases (I20–I25), acute myocardial infarction (I21–I22), other acute ischemic heart diseases (I24), other forms of chronic ischemic heart disease (I20, I25), other heart diseases (I26–I51), subarachnoid hemorrhage (I60), intracerebral and other intracranial hemorrhage (I61–I62), cerebral infarction (I63), stroke, not specified as hemorrhage or infarction (I64), other cerebrovascular diseases and their sequelae (I67, I69) (19).

### Covariates

Information on gender (male, female), age (continuous), and ethnicity (whites, blacks, Mexican Americans, or others), education (college or higher, high school, or less than high school), the ratio of family income to poverty (> 3.50, 1.31–3.50, or ≤ 1.30), alcohol consumption, and smoking status (non-smoker, past smoker, or current smoker) were collected from the participants by using standardized questionnaires. Current alcohol intake was categorized as non-drinker (0 g/day), moderate drinker (< 28 g/day for men and < 14 g/day for women), and heavy drinker (≥ 28 g/day for men and ≥ 14 g/day for women) (20). For physical activity, inactive group was defined as those with reported no leisure-time physical activity. Active group was defined as those with self-reported leisure-time moderate activity of five or more times per week or leisure-time vigorous activity of three or more times per week. Those who were not inactive and did not meet the criteria for recommended levels of physical activity was defined as insufficiently active group (21). Healthy Eating Index−2010 (HEI−2010) reflected the sum total of 10 diet components (consumption of foods from the grain, fruit, vegetable, dairy, and meat food groups; intake of dietary fats, saturated fats, cholesterol, and sodium; and a variety score) and provided a measure of overall quality of an individual's diet. The score is from 0 to 100, with 100 being the best-quality diet (22). Total energy intake (TEI) was calculated using the U.S. Department of Agriculture Automated Multiple-pass Method. Body weight and height were used to calculate body mass index (BMI). Diabetes duration was categorized into < 1 year, from 1 to ≤ 4 years, from ≥ 5 to ≤ 9 years, and ≥10 years. Treatment of diabetes was further classified as no treatment, only oral hypoglycemic medication, only insulin treatment, and oral medication and insulin treatment. Fasting serum was obtained via venipuncture by a trained nurse. Blood biomarkers, including vitamin B12, glycated hemoglobinA1c (HbA1c), insulin, fasting glucose, and C-reactive protein (CRP), were also measured. Insulin resistance was assessed using homeostasis model assessment-insulin resistance (HOMA-IR; [fasting insulin × fasting glucose]/22.5). Dietary supplements of folic acids were provided by NHANES Dietary Supplements section. Estimated glomerular filtration rate (eGFR) details (23) were described in the Supplementary Table 1.

In the baseline interview, the participants were asked “Have you ever been told by a doctor that you had one or more of the following general medical illnesses: asthma, arthritis, cancer, chronic bronchitis, diabetes, hypertension, gout, lupus, stroke, heart disease, or thyroid disease?” Hypertension was defined as currently taking prescribed medication and/or systolic blood pressure level ≥ 140 mmHg and/or diastolic blood pressure level ≥ 90 mmHg. History of CVD and cancer was assessed from the answers to this question.

### Statistical Analysis

Appropriate sampling weights were used to reconstitute data on a representative population level for the entire U.S. due to the complex sampling design adapted by NHANES (24). The means and proportions of baseline characteristics were compared by using linear regression for continuous variables and logistic regression for categorical variables. We examined the cross-sectional association between RBC folate levels and diabetes prevalence by logistic regression model (PROC SURVEYLOGISTIC). Models were successively adjusted for gender, age, ethnicity, education, family income level, smoking status, alcohol intake, physical activity, TEI, HEI-2010, BMI, history of hypertension, dyslipidemia, CVD, cancer, dietary folate intake and CRP.

We applied multivariable Cox regression models (PROC SURVEYPHREG) to further examine the temporal associations of RBC folate levels with all-cause and cause-specific mortality in diabetes and non-diabetes, respectively, and hazard ratios (HRs) and 95% confidence intervals (CIs) were calculated. We treated the lowest quartile of RBC folate (< 121 ng/mL) as the reference group, and adjusted for succeeding confounders. Model 1 was adjusted for gender, age, ethnicity, education, family income level, smoking status, alcohol intake, physical activity TEI and HEI-2010. Model 2 was additionally adjusted for BMI, history of hypertension, dyslipidemia, CVD and cancer, diabetes medication use. Missing values of covariates were included in the model treated as dummy variables.

Stratified analyses were also conducted by age (< 60 or ≥ 60 years), sex (male or female), BMI (< 30.0 or ≥ 30.0 kg/m^2^), race/ethnicity (White or non-White), educational level (less than high school, high school, college or higher), ratio of family income to poverty ( ≤ 1.30, 1.31–3.50 or > 3.50), smoking status (never/former or current smokers), alcohol intake (none/moderate or heavy), physical activity (inactive/insufficiently active or active), history of cardiovascular disease (yes or no), history of hypertension (yes or no), history of cancer (yes or no), diabetes duration (< 5 years or ≥ 5 years), and treatment for diabetes (none or yes). The joint test was used to obtain a *P* value for interaction to examine the statistical significance of the difference between subgroups. We performed several sensitivity analyses to examine the robustness of our results. First, we noted that diabetes medication use, hypertensive medication use and blood vitamin B12 were closed associated with the risk of CVD mortality, and RBC folate concentration was influenced by dietary folate intake. To prevent overadjustment, we adjusted these factors in sensitivity analyses instead of the main analyses. Second, homocysteine concentrations were reported to possibly have a strong relationship with folate levels (25), thus, further adjustment was made for homocysteine in diabetes [data were only available in NHANES (1991–1994), *n* = 1244]. Third, we performed a sensitivity to analysis the association between RBC folate and mortality in patients who used diabetes drugs. Forth, we also excluded patients with cancer diseases, and the analyses were rerun. Fifth, to minimize potential reverse causation bias, participants who died within 4 years of follow-up were excluded, and we reran the analyses.

Model assumptions were checked for all the analyses between January 12, 2021, and May 27, 2021. All statistical analyses were conducted using SAS version 9.4 (SAS Institute, USA), and a two-sided *p* value < 0.05 was considered statistically significant.

## Results

### Baseline Characteristics

A total of 15,514 participants (mean age of 45 ± 0.5 years, 7415 [47.8%] male) were included in the present analysis. Table 1 showed the baseline characteristics between diabetes (*n* = 2,972) and non-diabetes (*n* = 12,542), and the baseline characteristics according to RBC folate quartiles were shown in Supplementary Table 2. The participants with diabetes were more likely to be older, non-white, female, and less educated than those with no diabetes. They were also likely to have lower income, inactive physical activity, higher scores in HEI, and less TEI and less likely to be heavy drinkers and active smokers. In addition, they had a higher prevalence of CVD, hypertension, and cancer; more daily intake of folic acids from food; and higher HbA1c, insulin, HOMA-IR, fasting glucose, and CRP levels; and lower blood vitamin B12 and eGFR levels.

**Table 1:** Baseline characteristics of diabetes in comparison to non-diabetes aged 20+, NHANES III 1988–1994.

**Characteristics**	**Baseline status of diabetes and non-diabetes**			
	**Diabetes (*****n*** **= 2,972)**		**Non-diabetes (*****n*** **= 12,542)**	
	**No. (%)**	**SE**	**No. (%)**	**SE**
Age, mean, years	57.7	0.4	43.0	0.4
BMI, mean, kg/m^2^	28.9	0.2	26.1	0.1
Sex, n (%)				
Male	1367 (45.1)	1.5	6048 (49.2)	0.5
Female	1605 (54.9)	1.5	6494 (50.8)	0.5
Race/ethnicity, n (%)				
Whites	1151 (74.0)	1.6	5368 (77.4)	1.3
Blacks	843 (12.9)	1.0	3370 (10.0)	0.6
Hispanics	867 (5.3)	0.5	3293 (4.9)	0.4
Others	111 (7.8)	0.9	511 (7.7)	0.9
Educational level, n (%)				
Less than high school	1129 (21.1)	1.4	2638 (10.0)	0.6
High school	1259 (48.2)	1.6	5984 (46.6)	1.1
College or higher	584 (30.6)	2.0	3920 (43.4)	1.2
Ratio of family income to poverty, n (%)				
≤ 1.30	1003 (21.1)	1.5	3531 (16.1)	1.0
1.31–3.50	1118 (40.0)	1.2	5140 (43.1)	1.1
>3.50	506 (29.8)	1.8	2744 (34.7)	1.3
Unknown	345 (9.0)	0.9	1127 (6.1)	0.4
Smoking status, n (%)				
Never	1329 (40.8)	1.4	6252 (46.2)	0.8
Former	1040 (37.8)	1.3	2859 (24.1)	0.7
Active	603 (21.4)	1.0	3430 (29.7)	0.9
Alcohol intake, n (%)				
None	2438 (79.9)	1.7	9419 (71.9)	1.3
Moderate	151 (5.2)	0.5	1093 (9.2)	0.6
Heavy	238 (10.7)	1.3	1639 (15.9)	0.8
Unknown	145 (4.1)	0.7	391 (3.0)	0.6
Physical activity, n (%)				
Inactive	879 (22.6)	1.2	2563 (13.7)	0.8
Insufficient	1175 (41.9)	1.8	5310 (45.0)	0.9
Sufficient	918 (35.4)	1.9	4669 (41.3)	1.1
HEI-2010, mean	65.1	0.4	63.5	0.3
Total energy intake, mean, Kcal	1830.4	24.4	2249.5	21.4
CVD, n (%)	395 (10.1)	0.6	544 (2.8)	0.2
Hypertension, n (%)	1615 (48.5)	1.3	3164 (19.9)	0.8
Cancer, n (%)	292 (12.9)	0.9	1164 (7.9)	0.4
Duration of diabetes, n (%)				
<1 year	1840 (66.7)	1.3	-	-
1–5 years	364 (11.6)	0.8	-	-
5–10 years	227 (7.3)	0.9	-	-
≥10 years	541 (14.3)	0.8	-	-
Treatment for diabetes, n (%)				
None	2057 (73.6)	1.4	-	-
Only Insulin	542 (15.6)	1.1	-	-
Only Pills	323 (9.6)	0.9	-	-
Pills and insulin	50 (1.3)	0.2	-	-
Dietary folate intake, mean, mcg	282.2	6.4	289.3	3.3
HbA1c, mean, %	6.6	0.1	5.2	0.04
GLU, mean, mg/dl	136.3	2.3	92.9	0.2
Insulin, mean, uU/mL	20.9	1.1	9.7	0.2
HOMA-IR, mean	8.6	0.9	2.3	0.04
Vitamin B12, mean, mcg	4.9	0.2	5.3	0.2
CRP, mean, mg/dL	0.6	0.01	0.4	0.03
eGFR, mean, mL/min per 1.73 m^2^	66.0	0.6	77.7	0.4

*Data were expressed as the mean (SD) or n (%)*.

*BMI, body mass index; HEI, Healthy Eating Index; CVD, cardiovascular disease; HbA1c, glycosylated hemoglobin; GLU, fasting glucose; HOMA-IR, homeostasis model assessment-insulin resistance; CRP, C-reactive protein; eGFR, estimated glomerular filtration rate*.

### Association Between RBC Folate and Diabetes

RBC folate concentration was categorized as quartile, and its association with the prevalence of diabetes was examined (Table 2). In Model 1 adjusted for age, sex, and race, the odds ratios (ORs) for diabetes were 1.55 (95% CI: 1.25–1.92), 1.60 (95% CI: 1.31–1.96), and 1.89 (95% CI: 1.48–2.41) for quartiles 2–4, respectively, compared with the lowest quartile of RBC folate. Further adjusting for potential confounders, the association remained robust, and those with the highest quartile of RBC folate had higher odds of diabetes (fully adjusted OR: 1.94 [95% CI: 1.53–2.48], P_trend_ < 0.001).

**Table 2:** Odds ratios of diabetes according to RBC folate status.

	**Odds ratio (95%CI) of prevalence of diabetes**				
	**Q1, < 121 ng/mL, *n* = 3,924**	**Q2, 122–161 ng/mL, *n* = 3,824**	**Q3, 162–225 ng/mL, *n* = 3877**	**Q4, ≥226 ng/mL, *n* = 3,889**	**P for trend**
Model 1	1 (ref.)	1.55 (1.25, 1.92)	1.60 (1.31, 1.96)	1.89 (1.48, 2.41)	<0.001
Model 2	1 (ref.)	1.63 (1.30, 2.03)	1.73 (1.39, 2.15)	2.10 (1.63, 2.71)	<0.001
Model 3	1 (ref.)	1.62 (1.30, 2.01)	1.70 (1.38, 2.09)	2.04 (1.58, 2.63)	<0.001
Model 4	1 (ref.)	1.60 (1.29, 1.98)	1.65 (1.34, 2.03)	1.94 (1.53, 2.48)	<0.001

*Data are presented as hazard ratio (95% confidence interval)*.

*Model 1: adjusted for age, sex and race/ethnicity*.

*Model 2: model 1 + education, family income level, smoking status, alcohol intake, physical activity, TEI and HEI-2010*.

*Model 3: model 2 + BMI, history of hypertension, history of dyslipidemia, baseline CVD and baseline cancer, treatment for diabetes and duration of diabetes*.

*Model 4: model 3 + CRP+ dietary folate intake*.

*TEI, total energy intake; HEI, Healthy Eating Index; BMI, body mass index; CVD, cardiovascular disease; CRP, C-reactive protein*.

### Association of RBC Folate With All-Cause and Cause-Specific Mortality

During 297,708 person–years of follow-up (median of 19.2 years), 6,106 total deaths occurred, including 1,867 deaths from CVD, 1,452 deaths from ischemic heart disease, and 415 deaths from stroke disease. In Cox regression analyses, diabetes with moderate and high RBC folate quartiles showed an increased hazard in all-cause and CVD mortality, especially in ischemic heart disease mortality (Model 1, Table 3). These associations remained robust after stepwise adjustment for confounders. In the fully adjusted model, the HRs and 95% CIs from the lowest to the highest quartile of RBC folate for diabetes were 1.00 (reference), 1.10 (0.86, 1.42), 1.12 (0.87, 1.43), and 1.30 (1.04, 1.63) for all-cause mortality; 1.00 (reference), 1.19 (0.74, 1.92), 1.73 (1.08, 2.76), and 1.47 (0.98, 2.22) for CVD mortality; 1.00 (reference), 1.25 (0.71, 2.21), 2.01 (1.19, 3.39) and 1.62 (1.05, 2.50) for ischemic heart disease mortality; 1.00 (reference), 0.85 (0.32, 2.23), 0.81 (0.34, 1.89) and 0.93 (0.40, 2.17) for Stroke Mortality (Model 2, Table 3). However, for non-Diabetes, a Mild Protective Effect Was Found in all-Cause Mortality, Ischemic heart disease mortality. In multiplicative interaction analysis, a significant interaction between RBC folate level and diabetes status was observed for CVD mortality (*P*_interaction_ = 0.0048), and ischemic heart disease mortality (*P*_interaction_ = 0.0022) and null for all-cause mortality (*P*_interaction_ = 0.057) and stroke mortality (*P*_interaction_ = 0.74).

**Table 3:** Association between RBC folate with all-cause and cause-specific mortality among 15,514 individuals.

	**RBC folate, ng/mL**									
	**Diabetes**					**Non-diabetes**				
	**<121 ng/mL,** ***n*** **= 507**	**122–161 ng/mL,** ***n*** **667**	**162–225 ng/mL,** ***n*** **748**	**≥226 ng/mL,** ***n*** **748**	**Per 100 ng/mL increment**	** <121 ng/mL,** ***n*** **3,417**	**122–161 ng/mL,** ***n*** **3,157**	**162–225 ng/mL,** ***n*** **3,079**	**≥226 ng/mL,** ***n*** **2,889**	**Per 100 ng/mL increment**
All-cause mortality										
Deaths/person years	317/8,066	405/11,266	533/12,667	729/14,391		909/71,446	898/65,690	968/62,101	1347/52,080	
Model 1	1 (ref.)	1.14 (0.89, 1.45)	1.37 (1.09, 1.71)	1.46 (1.17, 1.82)	1.10 (1.04, 1.16)	1 (ref.)	0.93 (0.83, 1.04)	0.89 (0.80, 0.999)	0.97 (0.86, 1.10)	1.04 (0.99, 1.09)
Model 2	1 (ref.)	1.12 (0.87, 1.43)	1.3 (1.04, 1.63)	1.31 (1.04, 1.67)	1.07 (1.02, 1.13)	1 (ref.)	0.94 (0.84, 1.04)	0.89 (0.80, 0.99)	0.97 (0.87, 1.09)	1.03 (0.99, 1.07)
P for interaction	0.057									
CVD mortality										
Deaths/person years	91/8,066	114/11,266	181/12,667	243/14,391		249/71,446	280/65,690	307/62,101	402/52,080	
Model 1	1 (ref.)	1.22 (0.80, 1.86)	1.83 (1.18, 2.84)	1.67 (1.14, 2.45)	1.08 (1.01, 1.15)	1 (ref.)	0.97 (0.74, 1.28)	0.97 (0.69, 1.35)	0.83 (0.62, 1.12)	0.98 (0.90, 1.05)
Model 2	1 (ref.)	1.19 (0.74, 1.92)	1.73 (1.08, 2.76)	1.47 (0.98, 2.22)	1.04 (0.97, 1.11)	1 (ref.)	0.98 (0.75, 1.29)	0.95 (0.69, 1.31)	0.82 (0.63, 1.08)	0.96 (0.90, 1.03)
P for interaction	0.0048									
Ischemic heart disease mortality										
Deaths/person years	67/8,066	83/11,266	148/12,667	199/14,391		202/71,446	210/65,690	228/62,101	315/52,080	
Model 1	1 (ref.)	1.30 (0.80, 2.12)	2.16 (1.33, 3.51)	1.87 (1.25, 2.79)	1.09 (1.01, 1.17)	1 (ref.)	0.86 (0.64, 1.16)	0.82 (0.55, 1.21)	0.73 (0.53, 0.996)	0.96 (0.88, 1.05)
Model 2	1 (ref.)	1.25 (0.71, 2.21)	2.01 (1.19, 3.39)	1.62 (1.05, 2.50)	1.06 (0.98, 1.14)	1 (ref.)	0.88 (0.65, 1.19)	0.81 (0.56, 1.17)	0.73 (0.54, 0.98)	0.95 (0.88, 1.03)
P for interaction	0.0022									
Stroke mortality										
Deaths/person years	24/8,066	31/11,266	33/12,667	44/14,391		47/71,446	70/65,690	79/62,101	87/52,080	
Model 1	1 (ref.)	0.87 (0.34, 2.23)	0.82 (0.35, 1.92)	1.02 (0.44, 2.34)	1.03 (0.86, 1.22)	1 (ref.)	1.70 (0.84, 3.42)	2.03 (1.14, 3.61)	1.55 (0.79, 3.04)	1.01 (0.88, 1.16)
Model 2	1 (ref.)	0.85 (0.32, 2.23)	0.81 (0.34, 1.89)	0.93 (0.40, 2.17)	0.99 (0.83, 1.18)	1 (ref.)	1.65 (0.84, 3.26)	1.92 (1.11, 3.31)	1.46 (0.78, 2.74)	0.99 (0.86, 1.14)
P for interaction	0.74									

*Values are n or hazard ratio (95% confidence interval) and are weighted except No. of deaths/ person years*.

*Model 1: adjusted for age, sex and race/ethnicity, education, family income level, smoking status, alcohol intake, physical activity, TEI and HEI-2010*.

*Model 2: model1+ BMI, history of hypertension, history of dyslipidemia, baseline CVD and baseline cancer, diabetes medication use. P for interaction is only for model 2*.

*TEI, total energy intake; HEI, Healthy Eating Index; BMI, body mass index; CVD, cardiovascular disease*.

### Sensitivity Analyses

In stratified analyses, the associations of RBC folate quartiles with all-cause and CVD mortality were robust across the strata of age, sex, BMI, race/ethnicity, educational level, ratio of family income to poverty, smoking status, alcohol intake, physical activity, CVD history, hypertension history, cancer history, diabetes duration, and diabetes treatment, and all the interaction terms were not significant (*P*_interaction_ > 0.001, Supplementary Table 3). Further sensitivity analyses with additional adjustment of diabetes medication use, hypertensive medication use, dietary folate intake and blood vitamin B12 level produced similar results (Supplementary Table 4). The association between RBC folate quartiles and all-cause, CVD, and ischemic heart disease mortality remained the same with further adjustment of serum homocysteine (Supplementary Table 5). Further sensitivity analysis had been conducted with diabetes medication use (Supplementary Table 6) and cancer patients excluded (Supplementary Table 7), and the results remained robust.

In addition, participants who died within 4 years of follow-up were excluded, and the associations were re-examined. The results did not alter the significance of the associations between RBC folate quartiles and all-cause and cause-specific mortality (Supplementary Table 8).

## Discussion

In this nationwide prospective cohort study, high levels of RBC folate were found to be significantly associated with an elevated risk of CVD and all-cause mortality in patients diagnosed with diabetes. Conversely, high levels of RBC folate were negatively associated with all-cause and ischemic heart disease mortality in non-diabetes. The findings highlighted that the relationship between folate status and adverse outcomes may differ by baseline diabetes status.

Previous studies showed that folic acid may have CVD benefits for the general population. One cohort study suggested a 69% increased risk of mortality from coronary heart disease among participants with the lowest serum folate level category (< 3 ng/mL) compared with those with the highest category (> 6 ng/mL) (26). Compared with men whose serum folate concentrations were in the lowest tertile (< 8.4 nmol/L), those whose concentrations were in the highest tertile (>11.3 nmol/L) had a factor-adjusted relative risk of acute coronary events of 0.35 (95% CI: 0.17, 0.73) (27). A meta-analysis also found a 4% lower risk of overall CVD with folic acid supplementation (the dosage of folic acid in the intervention groups ranged from 0.5 to 15 mg/day) (8). However, the association between folate levels and CVD outcomes in different metabolic populations was inconsistent. In patients after acute myocardial infarction, a trend toward an increased risk of recurrent cardiovascular disease (relative risk of 1.22 [95% CI: 1.00–1.50]) was observed in the group with 0.8 mg of folic acid, 0.4 mg of vitamin B12, and 40 mg of vitamin B6 daily (28). Compared with placebo, active treatment (supplements combining folic acid and vitamins B6 and B12) did not significantly decrease the risk of death from cardiovascular causes in patients who had vascular disease or diabetes (relative risk of 0.96 [95% CI: 0.81–1.13]) (25). A limited study showed that the association between folate and mortality may differ by diabetes status (29). A study from the U.S. indicated higher RBC folate quartiles (≥ 634 nmol/L for men and ≥ 659 nmol/L for women) were significantly associated with the risk of death among patients with diabetes (HRs of 2.81 [95% CIs 1.48–5.32]) (30), consistent with the findings of the present study.

Limited literature exists on the biological mechanism underlying the association between high RBC folate and increased mortality from CVD among patients with diabetes. Endothelial dysfunction was found to be critical to the pathogenesis of microvascular and macrovascular complications of diabetes (31), and damaged endothelium may enhance thrombogenicity (7). Despite a related report showing that folic acid supplementation provided beneficial effects on endothelial functions (32), another report showed no evidence that folic acid could improve the markers of endothelial dysfunction or inflammation in patients with type 2 diabetes (33). High levels of RBC folate are cautiously suspected to possibly be detrimental to the endothelial function of patients with diabetes, thus warranting further investigation. The association between folate and accurate DNA synthesis and cell division under pathological conditions may help explain the observed results in the present study. In one-carbon metabolism, folate (as a critical cofactor) played an important function in biologic methylation and *de-novo* nucleotide synthesis pathways (34, 35). Some studies showed that adequate folate intake was crucial for accurate DNA synthesis and cell division for it could help sustain the normal patterns of DNA methylation and minimize DNA damage (35–37). However, a case-control study found that folate intake was inversely associated with promoter methylation of tumor suppressor and DNA repair genes in adenoma tissue specimens (38). Genetic polymorphisms of enzymes involved in one-carbon metabolism need to be considered when a complex picture of folate is being studied in the prevention of diseases (39). More observations should be conducted to investigate whether excessive folic acid may change promoter-specific methylation patterns in DNA or in histones among patients with diabetes.

Fortification of food with folic acid was introduced more than 20 years in North America to reduce the number of neural tube defects. Many countries are considering whether to adopt the same policy; however, little has been conducted at the population level to seriously trade-off the benefits to the few and the harm to some of the many exposed. Elderly people with high folate and low vitamin B-12 status had higher risk of cognitive impairment (OR: 5.1; 95% CI: 2.7, 9.5) and anemia (OR: 5.2; 95% CI: 2.5, 11.0) than those who had normal vitamin B-12 and normal folate concentrations (40). The Pune Maternal Nutrition Study showed that mothers with a combination of high blood folate and low vitamin B-12 concentrations during pregnancy had their children at greater risk of insulin resistance and higher blood folate concentration (41). Thus, whether high folate concentrations could impair normal folate function in different metabolic populations should be considered. The present study calls for more vigorous assessment on the relationship between high folate levels and the conditions beyond neural tube defect, especially among populations with diabetes.

This study has several strengths. NHANES provides a large-scale nationally representative sample that allows delineation of the relation in a wide array of population. The follow-up of over 19 years is favorable in providing sufficient statistical power to estimate the long-term association between baseline folate status and mortality. Another feature of this study is that baseline information was collected prior to fortification of folic acid in foods, which avoids bias caused by fortification of food with folic acid. Nevertheless, several limitations of this study should be considered. First, RBC folate was only assessed once at baseline, thus precluding the observations of folate changes with mortality risk. Second, although the study population was selected from a nationally representative sample, it excluded institutionalized patients. Diabetes, CVD diseases, and other chronic diseases are more common among institutionalized elderly patients. Third, given the ethnic disparity in the metabolism and blood level of folate, directly generalizing the findings to other ethnicities should be given caution, and the results should be replicated in other ethnic and racial groups. Fourth, given the nature of the observational design, residual confounders and unmeasured bias may have existed, and causal inferences should be cautious.

## Conclusion

In the nationally representative population of over 19 years of follow-up, increasing levels in RBC folate were independently associated with an increased risk of all-cause and CVD mortality among those diagnosed with diabetes, while high levels of RBC folate had a mild protective effect in non-diabetes. The findings highlighted that blood folate may play different roles in different disease conditions, thus requiring further investigation.

## Data Availability Statement

Publicly available datasets were analyzed in this study. This data can be found here: https://www.cdc.gov/nchs/nhanes/index.htm.

## Ethics Statement

The studies involving human participants were reviewed and approved by National Center for Health Statistics's Institutional Review Board. The patients/participants provided their written informed consent to participate in this study.

## Author Contributions

HX and XL analyzed, interpreted data, and drafted the first manuscript. HX conceived and designed the study. SC analyzed and interpreted data. PC and SG revised the manuscript. XH conceived, designed the study, and revised it critically for important intellectual content. YL conducted critical review and revision of the manuscript for important intellectual content. All authors meet authorship criteria and approved the final version of the manuscript to be published.

## Funding

HX is supported by Hubei Provincial Health Commission Joint Fund Projects (WJ2019H258). The funders had no role in the design of the study, in collection and analysis of the data, in the preparation and review of the manuscript, or in the decision to publish the final manuscript.

## Conflict of Interest

The authors declare that the research was conducted in the absence of any commercial or financial relationships that could be construed as a potential conflict of interest.

## Publisher's Note

All claims expressed in this article are solely those of the authors and do not necessarily represent those of their affiliated organizations, or those of the publisher, the editors and the reviewers. Any product that may be evaluated in this article, or claim that may be made by its manufacturer, is not guaranteed or endorsed by the publisher.
