# Supplementary material for: Folate Status and Mortality in US Adults With Diabetes: A Nationally Representative Cohort Study
Source: Front Cardiovasc Med. 2022 Apr 25;9:802247. doi: 10.3389/fcvm.2022.802247 (PMC9082538; doi:10.3389/fcvm.2022.802247)
Supplement: Supplementary file 1 [file Data_Sheet_1.docx]

**Supplementary Online Content**

Supplementary Table 1. Creatinine Equation

Supplementary Table 2. Baseline characteristics of 15514 patients according to RBC folate quartiles

Supplementary Table 3. Stratified analyses of the associations of per 100 ng/mL increment in RBC folate with all-cause and CVD mortality among 2972 diabetes

Supplementary Table 4. Sensitivity analyses of the associations of RBC folate with all-cause and cause-specific mortality among 2972 diabetes

Supplementary Table 5. Hazards of all-cause and cause-specific mortality with further adjustment of HCY among 1244 diabetes

Supplementary Table 6. Hazards of all-cause and cause-specific mortality among 915 diabetes with diabetes medication use

Supplementary Table 7. Association between RBC folate with all-cause and cause-specific mortality among 2680 Diabetes without cancer

Supplementary Table 8. Hazards of all-cause and cause-specific mortality after excluding participants who died within four years of follow-up among 2621 diabetes

Supplementary Figure 1. Flowchart of eligible population

**Supplement Table 1. Creatinine Equation**

| Basis of Equation and Sex | Serum Creatinine | Equation for Estimating GFR |
| --- | --- | --- |
| Female | ≤0.7 | 144 × (Scr/0.7)^−0.329^ × 0.993^Age^ [×1.159 if black] |
| Female | >0.7 | 144 × (Scr /0.7)^−1.209^ × 0.993^Age^ [× 1.159 if black] |
| Male | ≤0.9 | 144 × (Scr/0.9)^−0.411^ × 0.993^Age^ [× 1.159 if black] |
| Male | >0.9 | 144 × (Scr/0.9)^−1.209^ × 0.993^Age^ [× 1.159 if black] |

Abbreviations: Scr, Serum creatinine.

**Supplementary Table 2. Baseline characteristics of 15514 patients according to RBC folate quartiles**

| **Characteristics** | **RBC folate quartiles** | | | |
| --- | --- | --- | --- | --- |
|  | **Q1, n=3924 (<121ng/ml)** | **Q2, n=3824**  **(122-161ng/ml)** | **Q3, n=3877**  **(162-225ng/ml)** | **Q4, n=3889**  **(≥226ng/ml)** |
| **Age, mean (SD), years** | 40.2 (0.4) | 41.5 (0.5) | 44.8 (0.5) | 51.6 (0.8) |
| **BMI, mean (SD), kg/m2** | 26.5 (0.2) | 26.4 (0.1) | 26.7 (0.2) | 26.6 (0.2) |
| **Sex, n (%)** |  |  |  |  |
| **Male** | 1922 (49.4%) | 1942 (52.3%) | 1895 (51.6%) | 1656 (42.4%) |
| **Female** | 2002 (50.6%) | 1882 (47.7%) | 1982 (48.4%) | 2233 (57.6%) |
| **Race/ethnicity, n (%)** |  |  |  |  |
| **Whites** | 1039 (66.5%) | 1308 (71.9%) | 1702 (77.8%) | 2470 (87.4%) |
| **Blacks** | 1720 (20.7%) | 1103 (11.8%) | 879 (8.2%) | 511 (4.1%) |
| **Hispanics** | 1034 (5.8%) | 1233 (6.5%) | 1121 (5.1%) | 772 (3.0%) |
| **Others** | 131 (7.1%) | 180 (9.7%) | 175 (8.9%) | 136 (5.5%) |
| **Educational level, n (%)** |  |  |  |  |
| **Less than high school** | 932 (11.9%) | 1015 (12.5%) | 944 (11.6%) | 876 (10.5%) |
| **High school** | 2048 (56.1%) | 1783 (48.8%) | 1755 (44.0%) | 1657 (41.4%) |
| **College or higher** | 944 (31.9%) | 1026 (38.7%) | 1178 (44.4%) | 1356 (48.1%) |
| **Ratio of family income to poverty, n (%)** |  |  |  |  |
| **≤1.30** | 1428 (22.1%) | 1240 (18.9%) | 1067 (15.7%) | 799 (12.6%) |
| **1.31-3.50** | 1536 (45.2%) | 1527 (44.9%) | 1574 (41.4%) | 1621 (40.1%) |
| **>3.50** | 586 (25.9%) | 687 (29.5%) | 858 (36.3%) | 1119 (41.3%) |
| **Unknown** | 374 (6.7%) | 370 (6.7%) | 378 (6.6%) | 350 (6.0%) |
| **Smoking status, n (%)** |  |  |  |  |
| **Never** | 1768 (38.9%) | 1881 (44.7%) | 1909 (46.4%) | 2023 (49.8%) |
| **Former** | 666 (16.2%) | 848 (21.4%) | 1095 (28.7%) | 1290 (34.1%) |
| **Active** | 1489 (44.9%) | 1095 (33.9%) | 873 (24.9%) | 576 (16.0%) |
| **Alcohol intake, n (%)** |  |  |  |  |
| **None** | 3008 (76.0%) | 2887 (71.2%) | 2900 (69.9%) | 3062 (75.3%) |
| **Moderate** | 318 (7.7%) | 345 (10.2%) | 306 (9.2%) | 275 (7.6%) |
| **Heavy** | 460 (13.1%) | 459 (15.4%) | 533 (17.6%) | 425 (14.1%) |
| **Unknown** | 138 (3.2%) | 133 (3.2%) | 138 (3.4%) | 127 (3.0%) |
| **Physical activity, n (%)** |  |  |  |  |
| **Inactive** | 944 (16.6%) | 856 (16.7%) | 787 (12.4%) | 855 (14.7%) |
| **Insufficient** | 1711 (47.7%) | 1655 (46.3%) | 1667 (45.4%) | 1452 (40.1%) |
| **Sufficient** | 1269 (35.7%) | 1313 (36.9%) | 1423 (42.2%) | 1582 (45.2%) |
| **HEI-2010, mean (SD)** | 58.7 (0.4) | 61.7 (0.4) | 64.1 (0.3) | 68.5 (0.4) |
| **Total energy intake, mean (SD), Kcal** | 2209.6 (25.1) | 2266.5 (32.2) | 2243.8 (36.7) | 2068.7 (28.4) |
| **CVD, n (%)** | 176 (2.7%) | 181 (2.9%) | 248 (4.0%) | 334 (5.3%) |
| **Hypertension, n (%)** | 911 (17.4%) | 1031 (19.9%) | 1223 (23.4%) | 1614 (32.4%) |
| **Cancer, n (%)** | 177 (6.0%) | 176 (5.3%) | 297 (7.8%) | 514 (11.3%) |
| **Duration of diabetes, n (%)** |  |  |  |  |
| **<1 year** | 3779 (97.5%) | 3606 (97.2%) | 3556 (95.3%) | 3441 (92.1%) |
| **1-5 years** | 52 (1.1%) | 67 (1.0%) | 104 (1.7%) | 141 (2.5%) |
| **5-10 years** | 27 (0.3%) | 51 (0.6%) | 53 (1.2%) | 96 (1.9%) |
| **≥10 years** | 66 (1.2%) | 100 (1.2%) | 164 (1.9%) | 211 (3.5%) |
| **Treatment for diabetes, n (%)** |  |  |  |  |
| **None** | 3814 (98.5%) | 3654 (97.6%) | 3595 (95.8%) | 3536 (93.9%) |
| **Only Insulin** | 74 (1.0%) | 108 (1.6%) | 164 (2.4%) | 196 (3.5%) |
| **Only Pills** | 33 (0.5%) | 52 (0.6%) | 92 (1.5%) | 146 (2.5%) |
| **Pills and insulin** | 3 (0.1%) | 10 (0.2%) | 26 (0.3%) | 11 (0.1%) |
| **Dietary folate intake, mean (SD), mcg** | 222.6 (3.8) | 263.3 (4.1) | 300.1 (5.6) | 343.2 (5.5) |
| **HbA1c, mean (SD), %** | 5.4 (0.02) | 5.3 (0.03) | 5.4 (0.02) | 5.4 (0.04) |
| **GLU, mean (SD), mg/dl** | 95.2 (0.6) | 96.5 (0.6) | 100.0 (0.8) | 102.8 (1.0) |
| **Insulin, mean (SD), uU/mL** | 10.8 (0.3) | 10.7 (0.3) | 11.7 (0.5) | 11.7 (0.4) |
| **HOMA-IR, mean (SD)** | 2.7 (0.1) | 2.8 (0.1) | 3.2 (0.2) | 3.7 (0.4) |
| **Vitamin B12, mean (SD), mcg** | 5.0 (0.3) | 5.1 (0.2) | 5.4 (0.2) | 5.4 (0.2) |
| **CRP, mean (SD), mg/dL** | 0.4 (0.01) | 0.4 (0.01) | 0.4 (0.02) | 0.4 (0.02) |
| **eGFR, mean (SD), mL/min per 1.73 m2** | 80.8 (0.6) | 79.4 (0.6) | 76.9 (0.5) | 70.4 (0.5) |

Data were expressed as the mean (SD) or n (%).

Abbreviations: BMI, body mass index; HEI, Healthy Eating Index; CVD, cardiovascular disease; HbA1c, glycosylated hemoglobin; GLU, fasting glucose; HOMA-IR, homeostasis model assessment-insulin resistance; CRP, C-reactive protein; eGFR, estimated glomerular filtration rate.

**Supplementary Table 3. Stratified analyses of the associations of per 100 ng/mL increment in RBC folate with all-cause and CVD mortality among 2972 diabetes**

|  | **Per 100 ng/mL increment in RBC folate** | ***P* value for interaction** |
| --- | --- | --- |
| **All-cause mortality** |  |  |
| **Age, years** |  |  |
| <60 (n=1284) | 1.19 (1.02, 1.38) | 0.14 |
| ≥60 (n=1688) | 1.09 (1.04, 1.15) |  |
| **Sex** |  |  |
| Male (n=1367) | 1.09 (1.004, 1.17) | 0.86 |
| Female (n=1605) | 1.08 (1.03, 1.14) |  |
| **BMI, kg/m2** |  |  |
| <30.0 (n=1824) | 1.09 (1.01, 1.17) | 0.50 |
| ≥30.0 (n=1131) | 1.09 (1.03, 1.16) |  |
| **Race/ethnicity** |  |  |
| White (n=1151) | 1.08 (1.02, 1.15) | 0.15 |
| Non-White (n=1821) | 1.07 (0.97, 1.18) |  |
| **Educational level** |  |  |
| Less than high school (n=1129) | 1.14 (1.04, 1.26) | 0.06 |
| High school (n=1259) | 1.10 (1.05, 1.16) |  |
| College or higher (n=584) | 1.04 (0.94, 1.16) |  |
| **Ratio of family income to poverty** |  |  |
| ≤1.30 (n=1003) | 1.18 (1.06, 1.31) | 0.71 |
| 1.31-3.50 (n=1118) | 1.10 (1.03, 1.17) |  |
| >3.50 (n=506) | 1.03 (0.91, 1.16) |  |
| **Smoking status** |  |  |
| Never/former (n=2369) | 1.09 (1.04, 1.14) | 0.39 |
| Active (n=603) | 1.13 (0.96, 1.32) |  |
| **Alcohol intake** |  |  |
| None/moderate (n=2589) | 1.10 (1.04, 1.18) | 0.55 |
| Heavy (n=238) | 1.08 (0.96, 1.22) |  |
| **Physical activity** |  |  |
| Inactive/insufficient (n=2054) | 1.09 (1.02, 1.18) | 0.57 |
| Sufficient (n=918) | 1.09 (1.02, 1.16) |  |
| **History of cardiovascular disease** |  |  |
| Yes (n=395) | 1.30 (1.16, 1.46) | 0.0014 |
| No (n=2577) | 1.06 (1.01, 1.12) |  |
| **History of Hypertension** |  |  |
| Yes (n=1788) | 1.08 (1.02, 1.14) | 0.24 |
| No (n=1184) | 1.12 (1.02, 1.23) |  |
| **History of cancer** |  |  |
| Yes (n=292) | 1.06 (0.96, 1.17) | 0.63 |
| No (n=2680) | 1.10 (1.03, 1.17) |  |
| **Duration of diabetes** |  |  |
| <5 years (n=2204) | 1.08 (1.03, 1.14) | 0.98 |
| ≥5 years (n=768) | 1.06 (0.97, 1.16) |  |
| **Treatment for diabetes** |  |  |
| None (n=2057) | 1.07 (1.01, 1.14) | 0.90 |
| Yes (n=915) | 1.08 (1.01, 1.17) |  |
|  |  |  |
| **CVD mortality** |  |  |
| **Age, years** |  |  |
| <60 (n=1284) | 1.15 (0.85, 1.56) | 0.41 |
| ≥60 (n=1688) | 1.07 (0.99, 1.15) |  |
| **Sex** |  |  |
| Male (n=1367) | 0.99 (0.86, 1.12) | 0.52 |
| Female (n=1605) | 1.11 (0.999, 1.23) |  |
| **BMI, kg/m2** |  |  |
| <30.0 (n=1824) | 1.08 (0.97, 1.20) | 0.65 |
| ≥30.0 (n=1131) | 1.05 (0.95, 1.17) |  |
| **Race/ethnicity** |  |  |
| White (n=1151) | 1.07 (0.99, 1.15) | 0.97 |
| Non-White (n=1821) | 1.02 (0.84, 1.24) |  |
| **Educational level** |  |  |
| Less than high school (n=1129) | 1.18 (0.99, 1.40) | 0.05 |
| High school (n=1259) | 1.08 (0.95, 1.22) |  |
| College or higher (n=584) | 0.89 (0.76, 1.04) |  |
| **Ratio of family income to poverty** |  |  |
| ≤1.30 (n=1003) | 1.23 (1.09, 1.38) | 0.49 |
| 1.31-3.50 (n=1118) | 1.01 (0.87, 1.17) |  |
| >3.50 (n=506) | 0.99 (0.79, 1.24) |  |
| **Smoking status** |  |  |
| Never/former (n=2369) | 1.05 (0.97, 1.14) | 0.20 |
| Active (n=603) | 1.17 (0.98, 1.39) |  |
| **Alcohol intake** |  |  |
| None/moderate (n=2589) | 1.07 (0.98, 1.17) | 0.95 |
| Heavy (n=238) | 1.02 (0.73, 1.42) |  |
| **Physical activity** |  |  |
| Inactive/insufficient (n=2054) | 1.11 (1.01, 1.23) | 0.06 |
| Sufficient (n=918) | 0.93 (0.82, 1.06) |  |
| **History of cardiovascular disease** |  |  |
| Yes (n=395) | 1.30 (1.06, 1.60) | 0.06 |
| No (n=2577) | 1.02 (0.95, 1.10) |  |
| **History of Hypertension** |  |  |
| Yes (n=1788) | 1.03 (0.93, 1.14) | 0.16 |
| No (n=1184) | 1.21 (1.09, 1.34) |  |
| **History of cancer** |  |  |
| Yes (n=292) | 1.002 (0.81, 1.25) | 0.20 |
| No (n=2680) | 1.09 (1.02, 1.17) |  |
| **Duration of diabetes** |  |  |
| <5 years (n=2204) | 1.03 (0.94, 1.12) | 0.42 |
| ≥5 years (n=768) | 1.08 (0.94, 1.25) |  |
| **Treatment for diabetes** |  |  |
| None (n=2057) | 0.99 (0.90, 1.08) | 0.22 |
| Yes (n=915) | 1.13 (1.003, 1.27) |  |

Data were presented as hazard ratios (95% CIs) with adjustment of age, sex, and BMI, race/ethnicity, education, ratio of family income to poverty, smoking status, alcohol intake, physical activity, history of CVD, hypertension, cancer, duration and treatment of diabetes.

Abbreviations: BMI, body mass index; CVD, cardiovascular disease.

**Supplementary Table 4. Sensitivity analyses of the associations of RBC folate with all-cause and cause-specific mortality among 2972 diabetes**

|  | **RBC folate, ng/mL** | | | |
| --- | --- | --- | --- | --- |
|  | **<135ng/mL, n=740** | **136-182ng/mL, n=742** | **183-254ng/mL, n=748** | **≥255ng/mL, n=742** |
| **All-cause mortality** |  |  |  |  |
| Model 1 | 1 (ref.) | 1.17 (0.96, 1.43) | 1.37 (1.08, 1.73) | 1.39 (1.11, 1.74) |
| Model 2 | 1 (ref.) | 1.16 (0.94, 1.43) | 1.29 (1.03, 1.61) | 1.28 (1.03, 1.60) |
| Model 3 | 1 (ref.) | 1.15 (0.93, 1.41) | 1.28 (1.02, 1.60) | 1.27 (1.02, 1.59) |
| Model 4 | 1 (ref.) | 1.13 (0.92, 1.39) | 1.26 (1.004, 1.58) | 1.25 (0.997, 1.56) |
| Model 5 | 1 (ref.) | 1.14 (0.93, 1.39) | 1.27 (1.02, 1.59) | 1.26 (1.01, 1.57) |
| **Cardiovascular disease mortality** |  |  |  |  |
| Model 1 | 1 (ref.) | 1.61 (0.99, 2.61) | 1.92 (1.24, 2.97) | 1.80 (1.18, 2.74) |
| Model 2 | 1 (ref.) | 1.61 (0.98, 2.63) | 1.81 (1.19, 2.76) | 1.67 (1.11, 2.50) |
| Model 3 | 1 (ref.) | 1.57 (0.96, 2.56) | 1.79 (1.17, 2.72) | 1.65 (1.10, 2.48) |
| Model 4 | 1 (ref.) | 1.61 (1.00, 2.59) | 1.84 (1.23, 2.75) | 1.70 (1.15, 2.51) |
| Model 5 | 1 (ref.) | 1.63 (1.01, 2.65) | 1.93 (1.29, 2.89) | 1.78 (1.20, 2.65) |
| **Heart disease mortality** |  |  |  |  |
| Model 1 | 1 (ref.) | 1.84 (1.04, 3.27) | 2.30 (1.40, 3.77) | 2.00 (1.26, 3.18) |
| Model 2 | 1 (ref.) | 1.85 (1.03, 3.32) | 2.15 (1.33, 3.48) | 1.84 (1.18, 2.88) |
| Model 3 | 1 (ref.) | 1.80 (1.01, 3.21) | 2.13 (1.32, 3.45) | 1.83 (1.16, 2.86) |
| Model 4 | 1 (ref.) | 1.89 (1.07, 3.31) | 2.25 (1.42, 3.58) | 1.94 (1.26, 3.00) |
| Model 5 | 1 (ref.) | 1.90 (1.07, 3.38) | 2.36 (1.49, 3.74) | 2.04 (1.32, 3.15) |
| **Stroke mortality** |  |  |  |  |
| Model 1 | 1 (ref.) | 0.93 (0.40, 2.19) | 0.86 (0.37, 2.00) | 1.22 (0.49, 3.03) |
| Model 2 | 1 (ref.) | 0.91 (0.39, 2.13) | 0.83 (0.35, 1.95) | 1.19 (0.48, 2.95) |
| Model 3 | 1 (ref.) | 0.88 (0.37, 2.10) | 0.80 (0.34, 1.87) | 1.16 (0.47, 2.89) |
| Model 4 | 1 (ref.) | 0.83 (0.33, 2.09) | 0.72 (0.31, 1.70) | 1.01 (0.38, 2.68) |
| Model 5 | 1 (ref.) | 0.88 (0.33, 2.30) | 0.78 (0.31, 1.96) | 1.11 (0.41, 3.02) |

Data were presented as hazard ratios (95% CIs).

Model 1: adjusted for age, sex and race/ethnicity, education, family income level, smoking status, alcohol intake, physical activity, TEI and HEI-2010, BMI, history of hypertension, history of dyslipidemia, baseline CVD and baseline cancer. Model 2: model1+ diabetes medication use.

Model 3: model2+ hypertensive medication use. Model 4: model3+ Vitamin B12. Model 5: model4+ dietary folate intake. Abbreviations: TEI, total energy intake; HEI, Healthy Eating Index; BMI, body mass index; CVD, cardiovascular disease.

**Supplementary Table 5. Hazards of all-cause and cause-specific mortality with further adjustment of HCY among 1244 diabetes**

|  | **RBC folate, ng/mL** | | | |
| --- | --- | --- | --- | --- |
|  | **<135ng/mL, n=311** | **136-184ng/mL, n=310** | **185-266ng/mL, n=313** | **≥267ng/mL, n=310** |
| **All-cause mortality** | 1 (ref.) | 1.24 (1.13, 1.37) | 1.62 (1.41, 1.86) | 1.51 (1.38, 1.64) |
| **CVD mortality** | 1 (ref.) | 2.60 (2.42, 2.79) | 3.30 (2.85, 3.83) | 2.32 (2.17, 2.48) |
| **Heart disease mortality** | 1 (ref.) | 3.16 (2.83, 3.52) | 3.67 (2.99, 4.49) | 2.72 (2.38, 3.10) |
| **Stroke mortality** | 1 (ref.) | 1.23 (0.85, 1.78) | 2.27 (1.54, 3.36) | 1.39 (1.10, 1.76) |

Data were presented as hazard ratios (95% CIs) with adjustment of age, sex and race/ethnicity, education, family income level, smoking status, alcohol intake, physical activity, TEI and HEI-2010, BMI, history of hypertension, history of dyslipidemia, baseline CVD and baseline cancer, HCY.

Abbreviations: TEI, total energy intake; HEI, Healthy Eating Index; BMI, body mass index; CVD, cardiovascular disease; HCY, serum homocysteine.

**Supplementary Table 6. Hazards of all-cause and cause-specific mortality among 915 diabetes with diabetes medication use**

|  | **RBC folate, ng/mL** | | | |
| --- | --- | --- | --- | --- |
|  | **<151ng/mL, n=230** | **152-197ng/mL, n=227** | **198-276ng/mL, n=230** | **≥277ng/mL, n=228** |
| **All-cause mortality** | 1 (ref.) | 1.69 (1.23, 2.33) | 1.32 (0.98, 1.77) | 1.37 (1.04, 1.82) |
| **Cardiovascular disease mortality** | 1 (ref.) | 2.61 (1.39, 4.90) | 2.09 (1.07, 4.06) | 2.28 (1.24, 4.18) |
| **Heart disease mortality** | 1 (ref.) | 3.14 (1.45, 6.81) | 2.28 (1.03, 5.08) | 2.36 (1.17, 4.77) |
| **Stroke mortality** | 1 (ref.) | 0.68 (0.21, 2.18) | 1.25 (0.46, 3.41) | 2.10 (0.47, 9.41) |

Data were presented as hazard ratios (95% CIs) with adjustment of age, sex and race/ethnicity, education, family income level, smoking status, alcohol intake, physical activity, TEI and HEI-2010, BMI, history of hypertension, history of dyslipidemia, baseline CVD and baseline cancer, diabetes medication use.

Abbreviations: TEI, total energy intake; HEI, Healthy Eating Index; BMI, body mass index; CVD, cardiovascular disease.

**Supplementary Table 7. Association between RBC folate with all-cause and cause-specific mortality among 2680 Diabetes without cancer**

|  | **RBC folate, ng/mL** | | | |
| --- | --- | --- | --- | --- |
|  | **<133ng/mL, n=667** | **134-179ng/mL, n=678** | **180-249ng/mL, n=669** | **≥250ng/mL, n=666** |
| **All-cause mortality** | 1 (ref.) | 0.68 (0.21, 2.18) | 1.25 (0.46, 3.41) | 2.10 (0.47, 9.41) |
| **Cardiovascular disease mortality** | 1 (ref.) | 1.63 (1.04, 2.55) | 1.65 (1.05, 2.60) | 1.63 (1.10, 2.41) |
| **Heart disease mortality** | 1 (ref.) | 1.79 (1.09, 2.95) | 1.91 (1.14, 3.21) | 1.74 (1.14, 2.65) |
| **Stroke mortality** | 1 (ref.) | 1.01 (0.42, 2.44) | 0.71 (0.33, 1.56) | 1.25 (0.61, 2.57) |

Data were presented as hazard ratios (95% CIs) with adjustment of age, sex and race/ethnicity, education, family income level, smoking status, alcohol intake, physical activity, TEI and HEI-2010, BMI, history of hypertension, history of dyslipidemia, baseline CVD and diabetes medication use.

Abbreviations: TEI, total energy intake; HEI, Healthy Eating Index; BMI, body mass index; CVD, cardiovascular disease.

**Supplementary Table 8. Hazards of all-cause and cause-specific mortality after excluding participants who died within four years of follow-up among 2621 diabetes**

|  | **RBC folate, ng/mL** | | | |
| --- | --- | --- | --- | --- |
|  | **<134ng/mL, n=660** | **135-181ng/mL, n=649** | **182-251ng/mL, n=657** | **≥252ng/mL, n=655** |
| **All-cause mortality** | 1 (ref.) | 1.13 (0.88, 1.44) | 1.32 (1.04, 1.68) | 1.28 (0.98, 1.67) |
| **CVD mortality** | 1 (ref.) | 1.83 (1.04, 3.22) | 1.90 (1.21, 2.97) | 1.77 (1.09, 2.89) |
| **Heart disease mortality** | 1 (ref.) | 2.14 (1.11, 4.13) | 2.40 (1.47, 3.93) | 1.98 (1.19, 3.30) |
| **Stroke mortality** | 1 (ref.) | 1.07 (0.47, 2.45) | 0.68 (0.28, 1.65) | 1.26 (0.48, 3.32) |

Data were presented as hazard ratios (95% CIs) with adjustment of age, sex and race/ethnicity, education, family income level, smoking status, alcohol intake, physical activity, TEI and HEI-2010, BMI, history of hypertension, history of dyslipidemia, baseline CVD and baseline cancer.

Abbreviations: TEI, total energy intake; HEI, Healthy Eating Index; BMI, body mass index; CVD, cardiovascular disease.

**
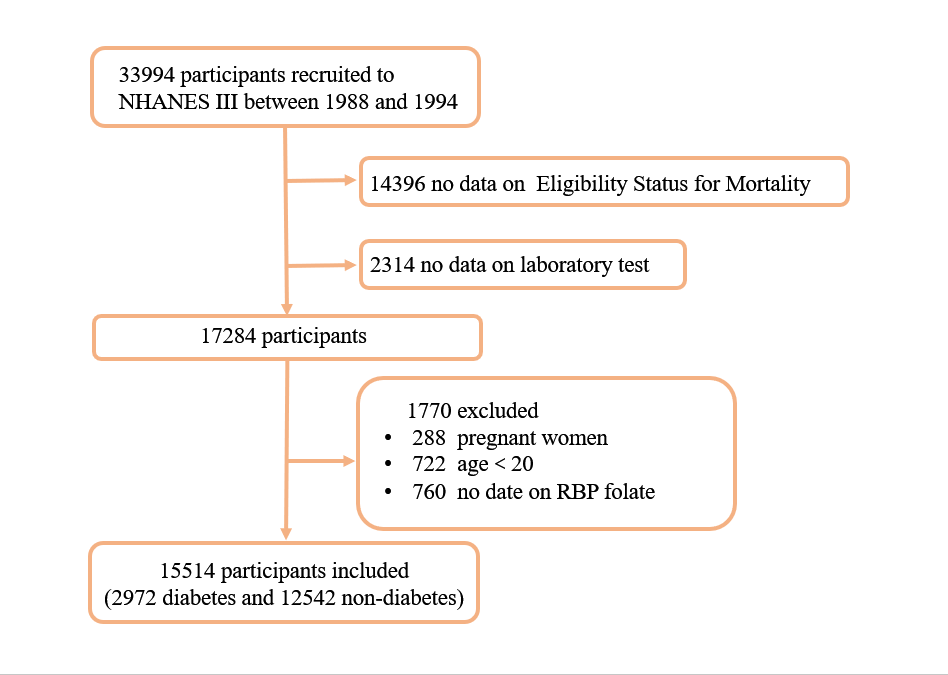
**

**Supplementary Figure 1. Flowchart of eligible population.**
